# Supplementary material for: Monitoring the Intracellular pH and Metabolic State of Cancer Cells in Response to Chemotherapy Using a Combination of Phosphorescence Lifetime Imaging Microscopy and Fluorescence Lifetime Imaging Microscopy
Source: Int J Mol Sci. 2023 Dec 19;25(1):49. doi: 10.3390/ijms25010049 (PMC10779161; doi:10.3390/ijms25010049)
Supplement: Supplementary file 1 [file ijms-25-00049-s001.zip › ijms-2708577-supplementary.pdf]

Supporting Information

SHG assay

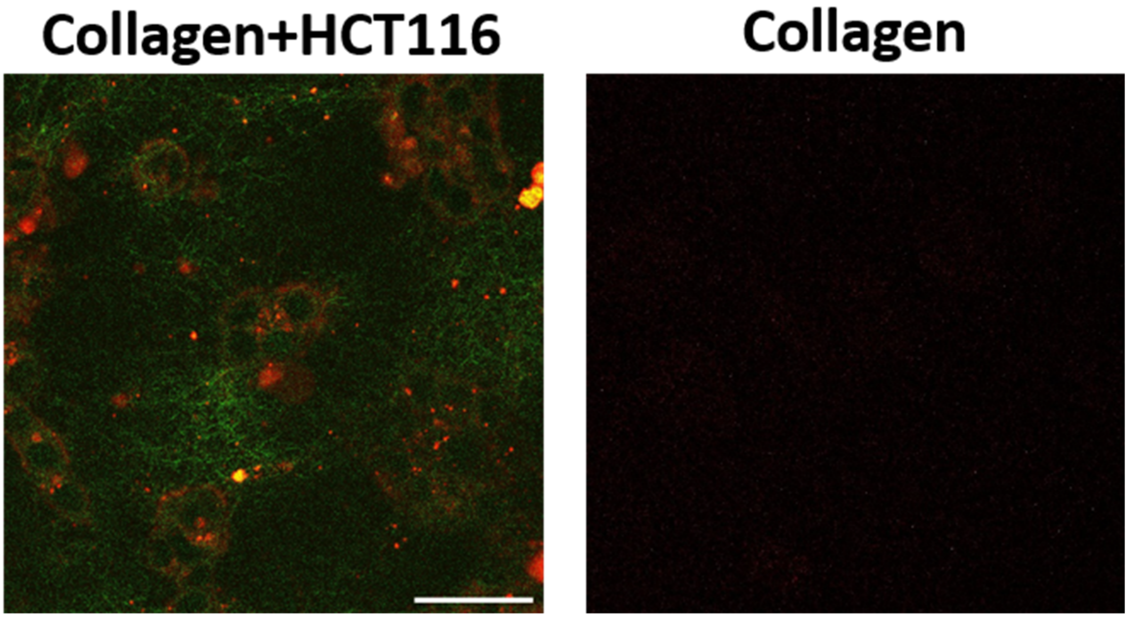

Figure S1. Representative SHG images of collagen seeded with HCT116 cancer cells or without any cells 24 h after seeding. Red - SHG signal from 327 collagen, green - autofluorescence of cells. Scale bar: 50 µm, applicable to all images

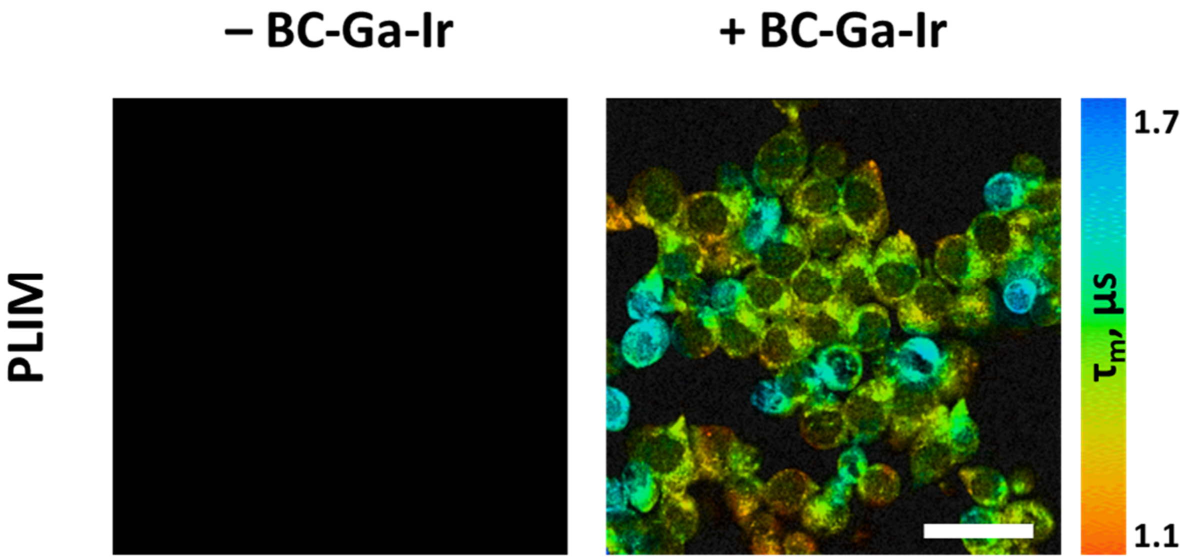

Figure S2. PLIM images of HCT116 tumor cells without adding a BC-Ga-Ir sensor and after 4 h of incubation with a BC-Ga-Ir sensor (concentration 10 µM). Scale bar = 50 µm.

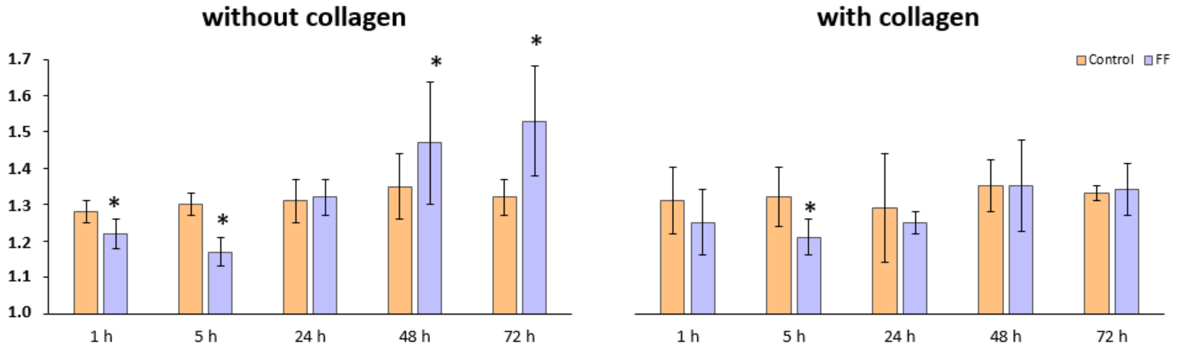

Figure S3. Phosphorescence lifetimes of the pHi-sensitive the BC-Ga-Ir probe in viable HCT116 cells cultured in the absence and presence of collagen. Mean ± SD. n = 30–40 cells. * $p \leq 0.05$ vs. controls.

Table S1. Phosphorescence lifetime of the BC-Ga-Ir probe in HCT116 cells in the model with and without collagen.

Median (Q1; Q3), * - difference from the control in one group (collagen, without collagen $p = 0.005$) (highlighted in red).

		**τ (1 h), µs**	**τ (5 h), µs**	**τ (24 h), µs**	**τ (48 h), µs**	**τ (72 h), µs**
Without collagen	Control	1.32 (1.29; 1.36)	1.29 (1.25; 1.31)	1.27 (1.25; 1.34)	1.34 (1.31; 1.38)	1.39 (1.34; 1.42)
	FF	1.24 (1.21; 1.27)*	1.12 (1.16; 1.21)*	1.30 (1.28; 1.34)	1.46 (1.42; 1.58)*	1.62 (1.53; 1.73)*
With collagen	Control	1.30 (1.25; 1.37)	1.24 (1.21; 1.32)	1.23 (1.26; 1.33)	1.31 (1.23; 1.37)	1.36 (1.33; 1.24)
	FF	1.23 (1.22; 1.33)	1.19 (1.15; 1.23)*	1.20 (1.14; 1.28)*	1.32 (1.26; 1.55)	1.38 (1.33; 1.43)

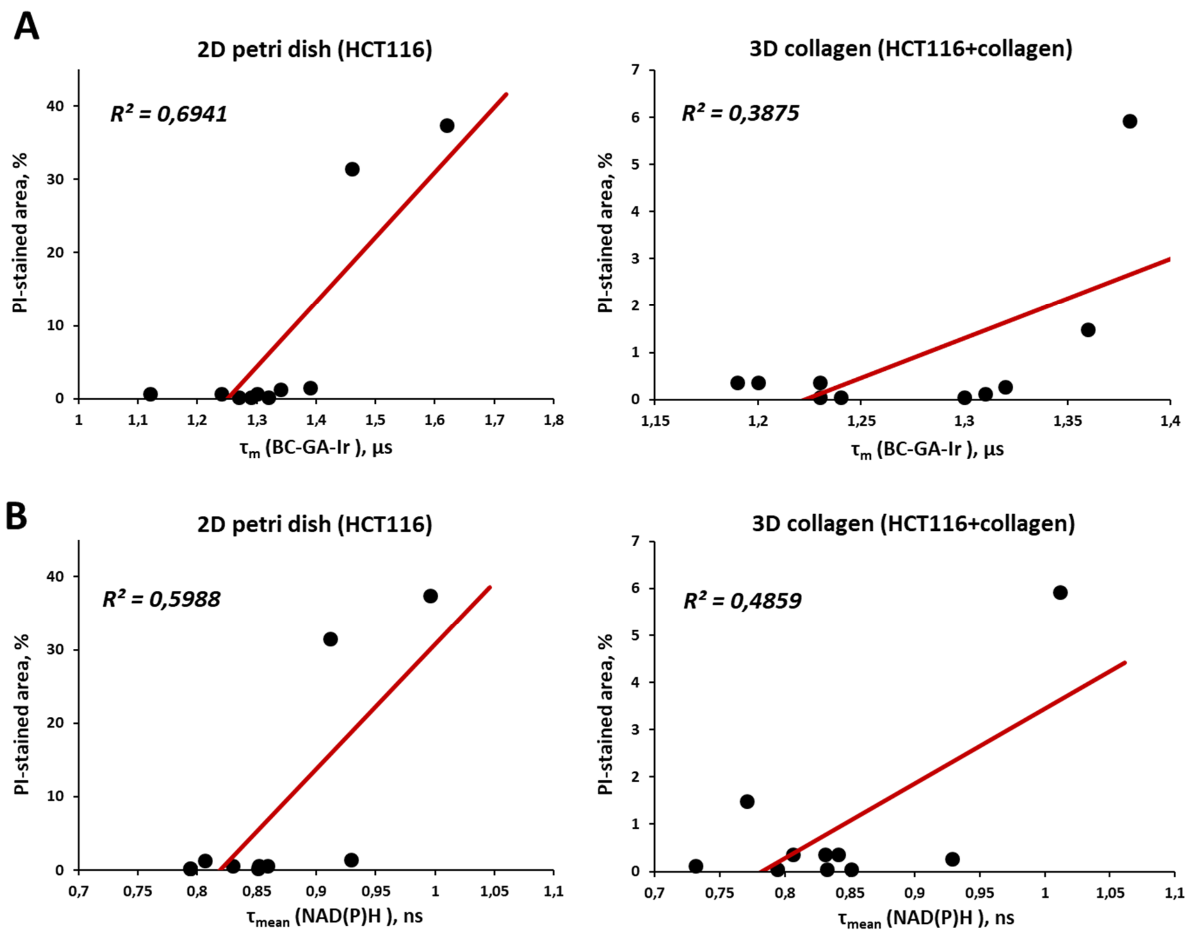

Figure S4. Analysis of correlation between pHi or metabolic state and cell viability. (**A**) Scatter plots for PI-staining (dead cells) and pHi measurements (τm BC-Ga-Ir) in 2D petri dish (HCT116) and 3D collagen culture (HCT116+collagen). (**B**) Scatter plots for PI-staining (dead cells) and metabolic measurements (τmean NAD(P)H) in 2D petri dish (HCT116) and 3D collagen culture (HCT116+collagen). Pearson correlation coefficients r are shown on the plots.

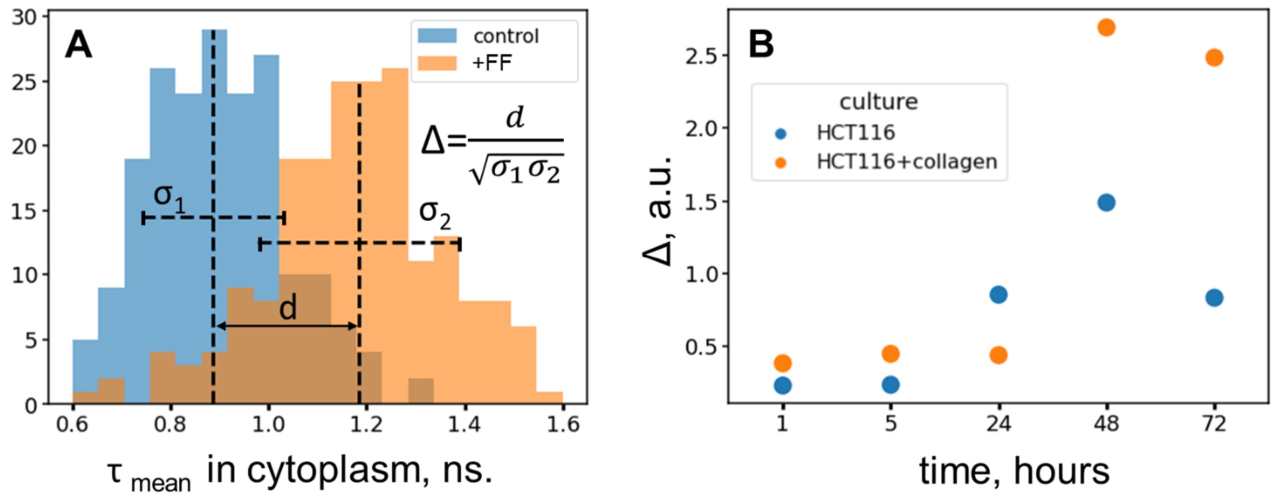

Figure S5. Comparison of the metabolic shift. (**A**) Determination of the delta parameter, changes in tau mean under the treatment of FOLFOX (**B**) Dependence of the delta parameter on the time of treatment to FOLFOX.

Comparison of metabolic differences

To compare the metabolic differences induced by FOLFOX treatment, we introduced the parameter Δ, defined as the ratio of the difference in the median values of the treatment and control samples to the mean standard deviation of the samples (see Figure S6).

After 24 hours of exposure, the effect is greater for a pure culture of HCT116 compared to a culture in collagen. At the same time, the maximum effect for both cultures is observed 48 hours after exposure to the drug.

Thus, for a culture with collagen, the effect of the FOLFOX treatment is slower compared to a pure culture, but more pronounced after 48 and 72 hours.

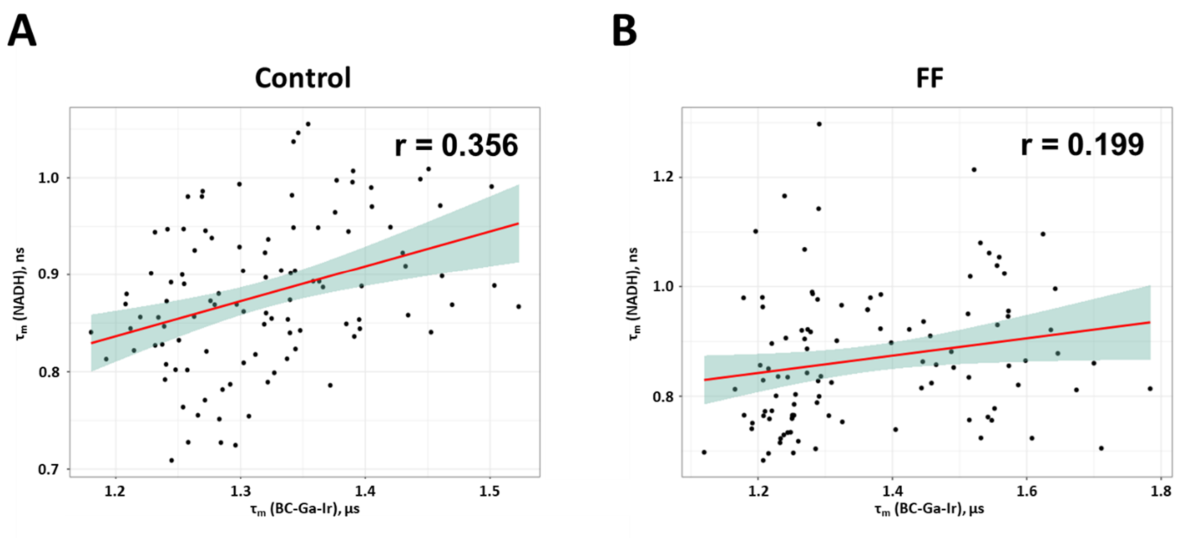

Figure S6. Analysis of correlation between metabolic state (NAD(P)H τ_{mean}) and pH (BC-Ga-Ir τ_m) at the single-cell level. Scatter plots for untreated control (**A**) and FOLFOX treated cells (**B**). Dots are the measurements for individual cancer cells in a 2D model. Pearson's correlation coefficients r are indicated on each plot. The solid red line represents the regression line.
